# Generating Comfortable Navigable Space for 3D Indoor Navigation Considering Users’ Dimensions

**DOI:** 10.3390/s20174964

**Published:** 2020-09-02

**Authors:** Wenjie Zhen, Lin Yang, Mei-Po Kwan, Zejun Zuo, Haoyue Qian, Shunping Zhou

**Affiliations:** 1 School of Geography and Information Engineering, China University of Geosciences, 388 Lumo Road, Wuhan 430074, China; zhenwenjie@cug.edu.cn (W.Z.); yanglin@cug.edu.cn (L.Y.); qhy_2015@cug.edu.cn (H.Q.); zhoushunping@mapgis.com (S.Z.); 2 Key Laboratory of Urban Land Resources Monitoring and Simulation, Ministry of Land and Resources, Shenzhen 518040, China; 3 State Key Laboratory of Geo-information Engineering, Xi’an 710054, China; 4 Department of Geography and Resource Management, and Institute of Space and Earth Information Science, Fok Ying Tung Remote Sensing Science Building, The Chinese University of Hong Kong, Hong Kong 780206, China; mpkwan@cuhk.edu.hk; 5 Department of Human Geography and Spatial Planning, Utrecht University, 3584 CB Utrecht, The Netherlands

**Keywords:** 3D indoor navigation, users’ dimensions, people-centric, pedestrian navigation, comfortable space

## Abstract

Most existing indoor navigation methods implicitly treat indoor users as ideal points. However, the ignorance of individual 3D indoor space needs may result in that navigation users do not have enough space or comfortable space to move in a real situation. Therefore, this paper proposes a novel human-oriented navigation approach that considers users’ dimensions and interactions with indoor objects to establish comfortable navigable space. First, object space (O-Space) for users is derived according to their types (i.e., non-disabled people or disabled people) and functional space (F-Space) for indoor objects is determined according to their functions, locations, sizes, and interactions. Then, narrow gaps where users cannot pass through easily are calculated based on indoor obstacles defined by O-Space, the use of F-Space, and stationary objects. Finally, comfortable navigable space is established by excluding inappropriate sealed spaces that wrap indoor obstacles and narrow gaps of the entire indoor space. Two indoor navigation cases were conducted and the results demonstrate that our method could provide comfortable space and user-friendly paths that navigation users can navigate easily without stress. Furthermore, our method also shows great potential for improving user experience during navigation, especially in unfamiliar indoor environments and even emergencies.

## 1. Introduction

Way-finding is a daily activity in our lives [1]. The demand for indoor navigation produced by the combination of Geographic Information System (GIS) and Building Information Model (BIM) has surged for user-friendly downstream applications. As users have higher needs for a comfortable and reliable navigation experience, establishing comfortable navigable spaces in people-oriented indoor navigation is an increasingly important research topic [2], which needs to consider not only the external environment (e.g., weather, noise) [3,4,5,6] but also the diversity of navigation user types. Although diversity (e.g., blindness, disability, obesity) has been studied a lot, user dimensions, specifically referring to the physical characteristics of users, have not been given the necessary attention so far in indoor environments [7,8,9]. Due to the compact and complicated characteristics of indoor environments (i.e., multiple floors, flexible spatial structures, and many indoor objects like tables and chairs) [10], an individual’s space needs in indoor navigation are much more closely related to user dimensions. Therefore, a customized and fine-grained indoor navigable space considering user dimensions may improve the user experience during navigation, especially in an unfamiliar environment or emergency [11,12].

To generate fine-grained indoor navigable space, various approaches are proposed to divide basic indoor space. A common approach is to use a dedicated strategy (e.g., visibility graph, Delaunay triangulation, and convex hull cell) or regular gridding (e.g., square, hexagon, etc.) to divide an entire indoor space for deriving a 2D navigable network [13,14,15,16,17]. To build complex indoor environments for pedestrian navigation [18,19,20], the 2D grid is expanded to the 3D grid (voxel). Unfortunately, with the exception of important architectural elements such as doors and stairs, very few studies pay special attention to the semantic information of indoor spaces until now. In regard to user dimensions, semantic context can provide useful environmental information (e.g., the impact of dynamic events such as fire diffusion, the detailed layout and the status of used indoor objects) to generate accurate navigable spaces for people-centric indoor navigation [17,21,22]. 

Thereafter, various semantic models are proposed by considering the details of indoor environments for the generation of navigable space [10,23,24]. First, several semantic space subdivision models in 2D space are developed. Afyouni et al. [25] established a fine-grained and multilayered spatial model, which uses a cell- and graph-based representation as the organizational structure of continuous indoor spaces and defines the navigability of the unit by considering indoor context information (including mobile users and continuous phenomena, e.g., fire diffusion). Besides, the functional space (F-Space) of the indoor object is proposed to represent the space where another object can physically interact with an object. Given the interactions between indoor objects in indoor space subdivisions, the granularity of indoor navigable 2D spaces is more refined. Kruminaite and Zlatanova [10] presented another framework, which determines the navigability of indoor spaces based on an indoor context at different times and the rules of human behavior, and then derives the navigation network by the constrained Delaunay triangulation of the navigable space. The notion of F-Space is further improved to characterize the interaction between indoor users and indoor objects, and the characteristics of the object (i.e., attractiveness, necessity, and closeness) and indoor users’ body projection are involved into the calculation of F-Space. Then, the semantic space subdivision models are expanded from 2D space to 3D space. Diakité and Zlatanova [26] provided a flexible space subdivision framework of indoor spaces (hereinafter referred to as FSS), which generates proper navigable space by considering various parameters (e.g., resources, agents, modifiers, and types of locomotion). The injection of abundant parameters enables FSS to identify the spaces available for indoor navigation and adapt to any 3D indoor configuration. For example, F-Space has been conceptually expanded from 2D space to 3D space, in which the object space (O-Space) and its generation method are proposed to define the space physically occupied by the object according to the dimensions of the object. The FSS framework has inspired fine-grained and context-aware indoor navigation and can play an illuminating role in future research on the generation of indoor navigable spaces.

Previous studies show that indoor navigable spaces have been further developed in 3D space to support more detailed granularity than in 2D space, especially fine-grained and context-sensitive indoor navigation frameworks. However, most existing studies have not paid much attention to 3D space dimensions of users (note that the users mentioned here are divided into two categories. One is the navigation user, namely “agent”. The other is the indoor people, excluding the navigation user, called “indoor user”. In this study, the term “users” is used to refer to these two categories). On the one hand, indoor users and indoor objects are the main participants in indoor space activities. The interaction between them will affect the navigability of the indoor space. As a typical subspace of a complex indoor environment, F-Space plays an important role in determining the accessibility of indoor spaces and it depends on the dimensions of indoor users to a large extent. Due to the lack of a detailed description of F-Space definition, the calculation of F-Space still depends on the body projection of the indoor user on the 2D plane [10], which limits its calculation in 3D space. On the other hand, most indoor navigation methods implicitly consider an agent as a 2D/3D point, which may result in agents not having enough space for the desired motion due to obstacles in complex indoor environments. 

In recent years, several studies have examined the dimensions of indoor navigators, especially wheelchair agents. To improve the safety of navigation, various solutions for smart wheelchairs have been proposed, which implicitly consider agents’ dimensions to avoid obstacles by processing data collected from different sensors (e.g., optical, infrared, and ultrasonic sensors) [27,28,29]. Geometrically, the American with Disabilities Act Accessibility Guidelines (ADAAG) provide detailed specifications by considering the required dimensions of wheelchair agents in indoor environments [30]. Various geometric methods are also developed to define inaccessible spaces for wheelchair agents (e.g., Minkowski sum and minimum distance (MD)) [31,32,33]. Kostic and Scheider [34] proposed a method for generating accessible space based on a grid model considering the shape of the agent and the wheelchair. Mantha et al. [35,36] simplified the navigation network by comparing the dimensions of the building structure and the dimensions of wheelchair agents. Liu and Zlatanova [37] presented an approach of calculating path for agents with different dimensions to avoid indoor obstacles in the 2D plane. Indoor obstacles based on the MDs between obstacles are grouped and the visibility graph is adopted to derive the indoor navigation network in 2D space. In 3D space, a similar approach is provided in the AccessVOR framework through a navigation network, which is composed of Voronoi edges of indoor obstacles to connect different floors [38]. However, these methods provide accessible indoor spaces from 2D views or rough 3D views, but still have limitations when describing complex 3D indoor environments in detail.

To address these limits, the primary aim of this paper is to fully consider users’ 3D dimensions in indoor environments to customize detailed and comfortable navigation paths for agents. This study pushes the generation framework of indoor navigable space forward (e.g., the FSS framework [26]), and explores the generation methods of user’s O-Space and indoor object’s F-Space by considering users’ 3D dimensions, and finally proposes a new method to establish comfortable navigable space (CN-Space) that allows users to pass comfortably without feeling stressed. 

Our work has made three innovative contributions: (1) By considering the dimensions of users in complex 3D space, the definition and generation method of O-Space for different types of users are proposed, which provides basic support for the generation of F-Space and CN-Space and (2) a new calculation method of the F-Space for indoor objects in complex 3D space is proposed based on new interactive rules. This method reidentifies the F-Space in use as non-navigable subspace and enriches the detailed expression of existing fine-grained indoor space subdivisions, which provides the possibility of accurately guiding agents in dynamic indoor environments. (3) The establishment of CN-Space can provide agents with humanized and effective navigation paths, which is especially important in emergencies. Meanwhile, CN-Space can also become an indicator of whether the indoor environment is friendly to agents. This enables people to improve the comfort of navigation services from the configuration of indoor space and demonstrates the true value of people-centric and fine-grained indoor navigation models and services.

The remainder of the paper is organized as follows. Section 2 elaborates on the generation method of CN-Space based on human perception of the environment and behavioral rules. In Section 3, the experiments are conducted to verify the validity of the proposed method by comparing it with the AccessVOR framework and the FSS framework. Finally, conclusions are provided in Section 4.

## 2. Method

### 2.1. Methodological Framework

**Figure 1:**
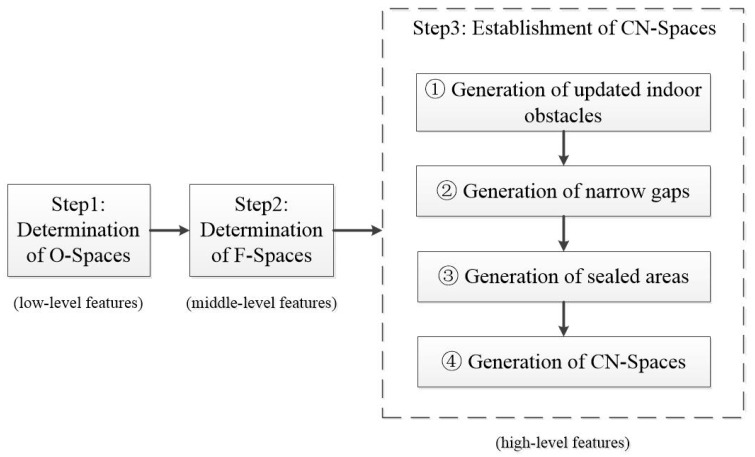
The flow chart of the generation process of CN-Spaces (comfortable navigable spaces).

In an indoor space, people often interact with each other and with various functional objects, which may affect the navigation of users. Therefore, three levels of semantics—geometric, functional, and human perceptual semantics—are introduced to define fine-grained indoor non-navigable subspaces that may affect users’ navigational comfort. The methodological framework for generating a CN-Space is divided into the following three main steps as shown in Figure 1: 

(1) **Determination of O-Space:** To simplify indoor environments, the spaces occupied physically by indoor objects that populate an indoor environment are defined as O-Spaces with coarse geometric representations [26]. In this paper, by considering the geometric features (low-level features) of users, the O-Spaces that are occupied physically by users’ bodies are detailly delineated and generated. All O-Spaces formed by indoor objects and indoor users are regarded as indoor obstacles.

(2) **Determination of F-Space:** The definition of F-Space is closely related to indoor users. In this paper, by considering the functional features (middle-level features) of indoor objects that are closely related to indoor users, the F-Spaces are generated. From the perspective of human behavioral rules, F-Spaces being used are also considered as indoor obstacles. 

(3) **Establishment of CN-Space:** Human perception in a 3D indoor environment (high-level features) is considered. In the vertical direction, spaces the agent cannot reach are regarded as non-navigable subspaces. Indoor obstacles are updated by removing these subspaces. In the horizontal direction, by the dimensions of the agent and identified indoor obstacles, narrow gaps where the agent cannot pass through easily are generated. Then sealed areas are created by indoor obstacles and narrow gaps. Based on the sealed areas and the location of the agent, new non-navigable subspaces are determined. Finally, to bypass these non-navigable subspaces, a CN-Space generation algorithm is designed to adapt to the existing navigation framework, which can meet agents’ needs for comfortable navigation. The following subsections provide detailed descriptions of the three steps. 

### 2.2. Determination of O-Spaces

In this study, to generate the O-Spaces of users, users are considered as a kind of special indoor object. The same operation performed for indoor objects in the Diakité and Zlatanova’s work [26] is performed for users. Namely, the smallest cuboid that encompasses a user is utilized to represent its user’s O-Space ($S_{o}$), which is defined as a 2-tuple in Equation (1).

(1) $$S_{o}=tuple\;\left(cuboid\;\langle position\rangle ,user\;dimensions\;\langle height,\;length,\;width\rangle \right)$$

where the height, length, and width of a user’s O-Space are first determined based on the orientation of the user. The length of the user’s front is defined as the length of individual O-Space. The width of the user’s side is defined as the width of individual O-Space. The height of the user is defined as the height of individual O-Space. 

**Figure 2:**
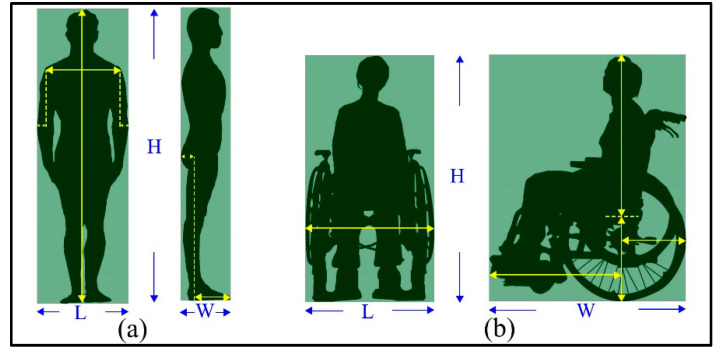
The object spaces (O-Spaces) of (**a**) a non-disabled adult and (**b**) a disabled adult.

Two types of users are considered: Non-disabled people and disabled people. Figure 2 shows two general examples of non-disabled people and disabled people, respectively. For non-disabled people, their O-Spaces are dependent on their bodies. The sizes of users’ bodies are related closely to their ages, race, and gender [39]. Compared to these factors, users’ clothing also has a small impact on the generation of their O-Spaces. The body of an adult standing naturally is shown in Figure 2a. The length of his O-Space is the sum of the width of his shoulders and a portion of the width of his elbows. Besides, the width of his O-Space is the sum of a portion of the width of his hips and a portion of the length of his feet. The height of his O-Space is his actual height. These analyses provide a promising way to use easily accessible data to obtain users’ O-Spaces. For example, there is an established relationship between height and various body parts like head, trunk, upper, and lower extremities [40]. However, these relationships may vary with age, race, and gender. Thus, based on regression analysis, other relevant body measurements could be acquired based on human height for different groups of people. Then, non-disabled users’ O-Spaces could be generated using these measurements. This method has been widely used in the determination of criminals and the dead in forensic examinations [39,41,42]. For disabled people, their O-Spaces are related to their bodies on the one hand and dependent on the specifications of their assistive devices on the other hand. Figure 2b shows the O-Space of a disabled adult in a wheelchair. The length and width of her O-Space are almost decided by the size of the wheelchair. The height of her O-Space is the sum of the length of her upper body and the height of the wheelchair cushion. Similar to the non-disabled, the O-Spaces of disabled users could also be generated by easily accessible data, such as human height, specifications of assistive devices, etc. After that, the O-Spaces of users can be analyzed and derived to facilitate the determination of the F-Space. 

### 2.3. Determination of F-Spaces

The determination of F-Spaces is complicated due to the diversity of the indoor objects’ functions. The generation method of F-Space has not been explicitly proposed in 3D space in previous studies. In this subsection, a generation method of F-Space is designed by considering indoor users’ O-Spaces and human behavioral rules.

**Figure 3:**
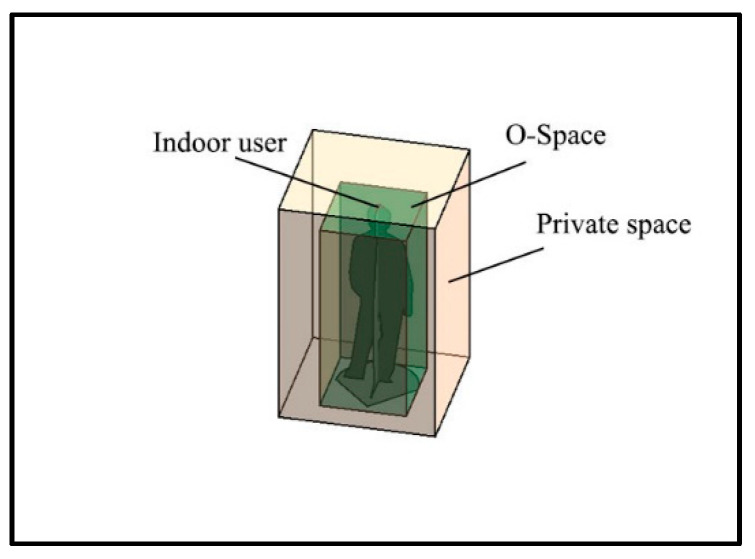
The O-Space and the private space of a user.

The private space is first introduced to generate F-Space [43]. The closer the user is to the obstacle, the more uncomfortable they feel when walking because they need to pay more attention to avoid injury [10,43,44]. Thus, for a user, there is a buffer space (i.e., private space) around the user’s O-Space to keep a certain separation distance from the wall, other users, or obstacles, as shown in Figure 3. The empirical separation distances in the vertical and horizontal directions are 0.45–1.2 m [43] or 0.4–0.8 m [45]. 

According to the determination of F-Space in 2D space [10] and the inspiration of the private space, F-Spaces of indoor objects should include two parts. One is the activity space of indoor users, namely, the space that indoor users need to interact with the object. Here, this study hypothesizes that the indoor user needs to be close to an indoor object to use it. In this way, the activity space should be at least as large as the O-Space of the indoor user. The other is the indoor users’ private space. When an indoor user is interacting with an object, he/she also needs to keep a separation distance from others to avoid being disturbed. Therefore, the F-Space of an object $S_{f}$ is determined by Equation (2):

(2) $$S_{f}=S_{o}+S_{private}\left(d\right)$$

where $S_{o}$ is the activity space of indoor users’ bodies and $S_{private}\left(d\right)$ is the private space of indoor users with the separation distance d.

**Figure 4:**
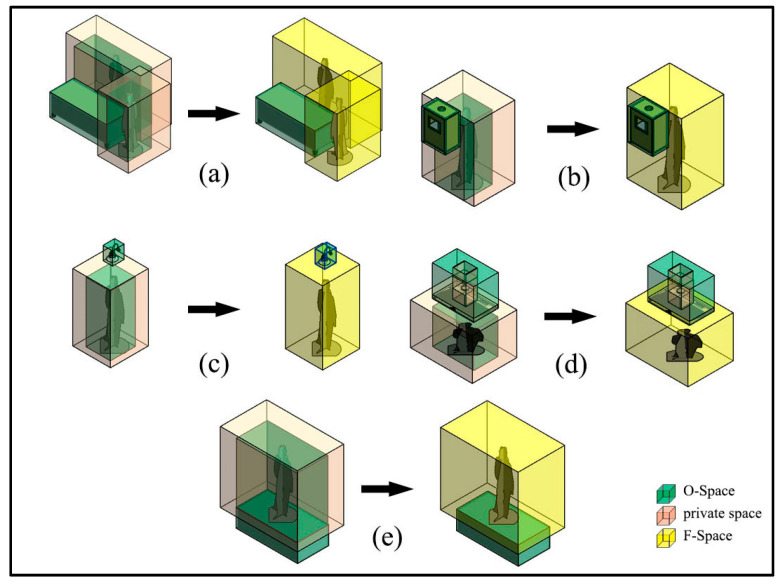
Examples of functional spaces (F-Spaces). (**a**) The F-Space of the table includes the back F-Space and the left F-Space. (**b**) The F-Space of a cabinet hanging on the wall. (**c**) The F-Space of a lamp. (**d**) The F-Space of the lamp close to the floor. (**e**) The F-Space of a mattress.

Based on the above definition, the F-Spaces of diverse indoor objects are determined in detail. Each O-Space (cuboid) has six faces (front, behind, left, right, top, and bottom). Due to the diversity of indoor objects, each face may become the interaction plane between the object and its indoor users. The generation of F-Space is related to the orientations of the faces and different for the six faces. For example, the faces around a table are the interaction planes, while the interaction plane for a weighing scale is the top face. Thus, optional F-Spaces for indoor objects need to be defined. F-Spaces of diverse indoor objects are determined using the following rules:

**Rule1**: In the top and bottom faces, the private space could be completely generated without limitation. Because it is unlimited in the horizontal direction, and from a top view, it can form a closed loop around the indoor user (see Figure 4c–e). In the other four faces, the private space is blocked by the O-Space of the object, because the O-Space of the indoor user is close to the O-Space of the object (see Figure 4a,b). 

**Rule2:** F-Space depends on the size of the O-Spaces of the object and its indoor users/user. If the length or width of the object’s O-Space is less than the length or width of the corresponding face of the indoor user, the indoor user’s O-Space is regarded as the activity space (see Figure 4b,c). Such objects are usually designed to be used by a single indoor user. Their F-Spaces can be built with a specific indoor user’s O-Space (when the object is private) or a preset generic indoor user’s O-Space (when the object is public). Otherwise, the indoor user’s O-Space expands so that its length or width is equal to the length or width of the O-Space of the object. The expanded space is regarded as the activity space (see Figure 4a,d,e). One or more indoor users can move into this activity space. Such objects are usually designed to be used by multiple indoor users, so their F-Spaces can be built with a preset generic indoor user’s O-Space. 

**Rule3:** The generation of the F-Space needs to consider the positions of the O-Space of the object and the indoor user. For the top face, the F-Space is unlimited in the vertical direction (see Figure 4e). However, for the other five faces, if the face that needs to generate the F-Space is higher than the top face of the private space of the indoor user (e.g., people use cabinets fixed to the wall), the indoor user who is standing on the floor may need to reach out or use a tool to interact with it. Hence, the F-Space is expanded in the vertical direction (see Figure 4b,c). For the bottom face, if the face is lower than the top face of the private space of the indoor user, the F-Space needs to be reduced in the vertical direction because of the limitation of the floor (see Figure 4d). Considering the above rules, the size of the F-Space is determined by Equations (3)–(5).

(3) $$L_{f}=max\left(L_{u},L_{o}\right)+2D_{private}$$

(4) $$W_{f}=\{\begin{array}{c} max\left(W_{u},W_{o}\right)+2D_{private}\;\;\;\;\;\;\left(for\;the\;top\;and\;bottom\;faces\right)\\ max\left(W_{u},W_{o}\right)+D_{private}\;\;\;\;\;\;\;\;\;\;\;\;\;\;\;\;\;\;\left(for\;the\;other\;four\;faces\right) \end{array}\;\;\;$$

(5) $$H_{f}=\left\{ \begin{array}{c} H_{u}+D_{private}\;\;\;\;\;\;\;\;\;\;\;\;\;\;\;\;\;\;\;\;\;\left(for\;the\;top\;face\right)\\ Height_{bottom}\;\;\;\;\;\;\;\;\;\;\;\;\;\;\;\;\;\;\;\;\;\;\;\;\;\;\;\;\left(for\;the\;bottom\;face\right)\\ max(H_{u}+D_{private},Height_{top})\;\;\;\;\;\;\;\;\;\;\left(for\;the\;other\;four\;faces\right) \end{array}\;\;\;\right.$$

where, $L_{f}$, $W_{f}$, and $H_{f}$ are the length, width, and height of the F-Space respectively. $L_{u}$, $W_{u}$, and $H_{u}$ are the length, width, and height of the O-Space of the indoor user, respectively. $L_{o}$, $W_{o}$, and $H_{o}$ are the length, width, and height of the O-Space of the object, respectively. Note that the positions of the length, width, and height of the F-Space and O-Space of the object correspond to the positions of the length, width, and height of the O-Space of the indoor user. $D_{private}$ is the buffer distance of the private space. $Height_{top}$ and $Height_{bottom}$ are the distances between the top and bottom of the O-Space of the object and floor, respectively.

### 2.4. Establishment of CN-Spaces

Based on the O-Spaces and F-Spaces defined above, this paper aims to provide adequate navigable space and generate a navigation path that the users can pass comfortably without stress. The navigation agent tends to keep a certain comfortable distance from the borders of buildings, walls, and other obstacles, therefore the design of CN-Space needs to allow the agent to face forward and pass corridors easily, avoid bending over, and turning sideways due to the lack of navigable space, especially for the blind [9]. A lot of indoor objects divide indoor space into subspaces with different sizes and subspaces that do not meet the requirements of adequate navigation space should be identified and deleted when constructing a CN-Space. Thus, indoor obstacles that the agent cannot reach in both vertical direction and horizontal direction should be defined first, and then narrow gaps that the agent cannot pass easily should be calculated. These two subspaces are both considered non-navigable subspaces. Finally, the CN-Space generation algorithm is designed based on non-navigable subspaces.

Indoor obstacles are first defined, which should include walls and indoor non-navigable subspaces. In Diakité and Zlatanova’s work [26], O-Space and F-Space are considered as non-navigable subspaces, namely indoor obstacles. However, not all F-Spaces are non-navigable. This study only qualifies that F-Spaces are not suitable for navigation or non-navigable when objects are interacting with their indoor users. In other words, the F-Spaces are navigable subspaces when objects are not in use and thus the available navigable space is expanded to some extent. Therefore, indoor obstacles IO are expressed as:

(6) $$IO=\left\{S_{o},S_{f_{u}},S_{w}\right\}$$

where, $S_{f_{u}}$ and $S_{w}$ are F-Spaces of the indoor objects interacting with their indoor users and walls, respectively. 

Based on the above definition, the establishment of CN-Space consists of three steps: (1) A subdivision is performed in the vertical direction and the spaces higher than the private space of the agent (including all IOs contained therein) are deleted. In this way, whether the agent can reach the remaining space comfortably in the horizontal direction is the only problem that needs to be considered. Thus, the problem is simplified from 3D space to a 2D plane. (2) On a 2D plane, the layout of IOs could create narrow gaps where the agent cannot pass through easily. To find these narrow gaps, the MDs between the IOs are analyzed and calculated. (3) IOs and narrow gaps are reconsidered from a 3D perspective to generate the CN-Space. These three steps are described in detail below.

**Figure 5:**
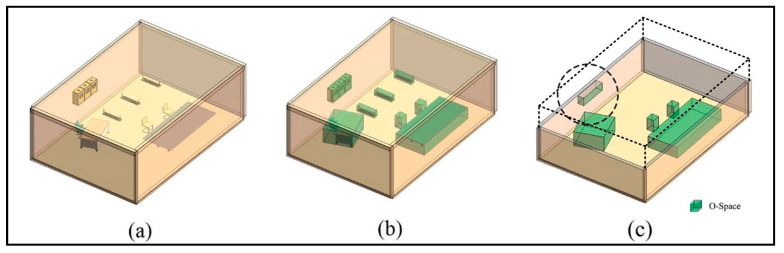
The layout of Room A. (**a**) Actual indoor environment. (**b**) Simplified indoor environment. (**c**) The indoor spaces after deleting $S_{h}$.

The procedures for the subdivision in the vertical direction are provided in Appendix A. Take Room A as an example to illustrate the algorithm, whose layout is shown in Figure 5a. This room includes a conference table, two chairs, a workbench, a row of cabinets, and three lamps. To simplify the indoor environment of Room A, O-Spaces of the indoor objects are generated to represent these subspaces occupied by them (Figure 5b). Since there is no interaction between human and indoor objects in this example, O-Spaces of the indoor objects and walls are used as IOs. The room and these O-Spaces are stored as cuboids in the database. Here, the bottom face (a series of lines end to end) and the maximum and minimum vertical heights $H_{up}$ and $H_{down}$ are used to describe a cuboid space. The question regarding which subspaces may affect the movement of the agent should be considered first. It is easy to understand that the agent relies on the floor all the time, so he/she cannot reach the higher subspaces above him/her which do not interfere with his/her movement. Thus, for a specified agent, in the vertical direction, the spaces higher than the private space of the agent (expressed as $S_{h}$) are deleted from the database (Figure 5c). The $H_{up}$ of the room is reset to $H_{private}$ (the height of the agent’s private space), and these IOs are updated. Note that $H_{down}$ of some IOs are lower than $H_{private}$ and their $H_{up}$ are higher than $H_{private}$. Hence, these IOs are only partially reserved, such as the part enclosed by the dotted circle in Figure 5c. 

**Figure 6:**
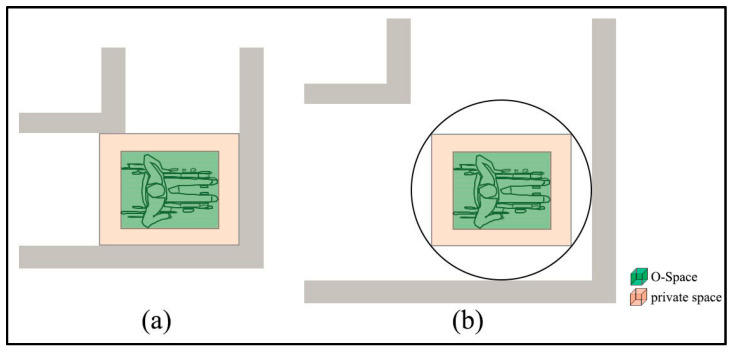
Determination of the buffer distance. (**a**) The width of the gap is the width of the agent’s private space. (**b**) The width of the gap is the diameter of the circumcircle.

Next, narrow gaps are generated based on the 2D projection of all IOs. At first, the IOs are projected onto the floor. Note that the walls are expressed as straight lines in the projection because their other planes do not participate in the indoor activities. Then, for each IO, a buffer operation is performed to find those surrounding IOs that may have narrow gaps with it. Each location in the CN-Space needs to not only meet the demand for accessibility but also take into account the fact that the agent could turn in any direction at any time. Therefore, the CN-Space must be large enough to accommodate the circumcircle of the projection of the agent’s private space. As shown in Figure 6, even if the width of the corridor allows the agent to walk straight, there is no guarantee that the agent will be able to turn comfortably at any time. In other words, the gaps in CN-Space must be larger than the circumcircle’s diameter of the projection of the agent’s private space. Therefore, the buffer distance is set to the diameter of the circumcircle and is calculated by Equation (7):

(7) $$D_{b}=\sqrt{L_{private}{}^{2}+W_{private}{}^{2}}$$

where $L_{private}$ and $W_{private}$ are the length and width of the agent’s private space, respectively. The procedures for the generation of narrow gaps are provided in Appendix B. 

**Figure 7:**
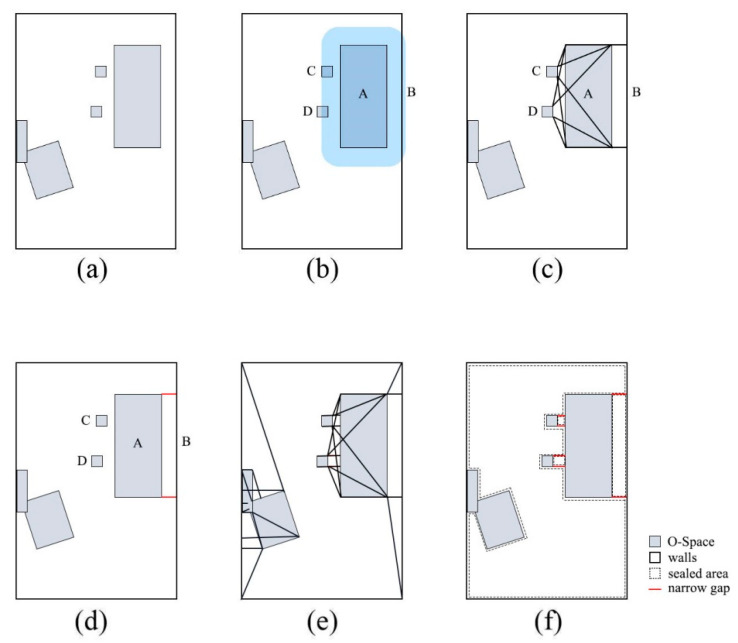
The generation of narrow gaps. (**a**) The projection of Room A. (**b**) The buffer of the IO A. (**c**) The minimum distances (MDs) between the endpoints of A and B, C, and D. (**d**) The narrow gaps of IO A. (**e)** All MDs between the endpoints of each IO and the IOs in its buffer. (**f**) The narrow gaps of Room A.

**Figure 8:**
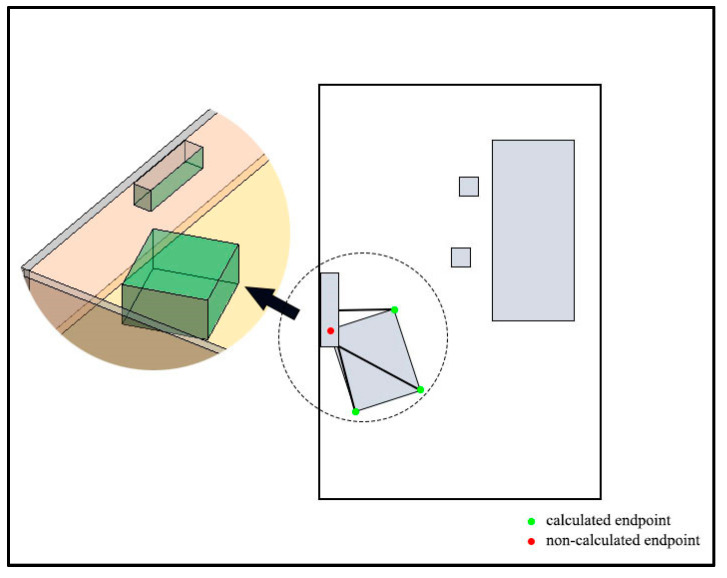
A special case that the projection of one endpoint of an IO is in the projection of another IO.

As shown in Figure 7, Room A is taken as an example to illustrate the algorithm in Appendix B. The projections of IOs in Room A are shown in Figure 7a. In Figure 7b, wall B and chairs C and D are included in the buffer of conference table A. The narrow gaps may be generated between any side of an IO and the nearby IOs. Even IOs that are not at the same height need to calculate their MDs in the horizontal direction, as the MD may affect the movement of the agent. Therefore, all the MDs between every side of an IO and every IO in its buffer need to be considered. In this study, the endpoints of IO are used instead of the sides to calculate these MDs. Figure 7c illustrates the MDs between each endpoint of IO A and IOs B, C, and D. A special case is shown in Figure 8. The projection of one endpoint of an IO is in the projection of another IO. In this case, the MDs between the endpoint and other surrounding IOs are not calculated. Meanwhile, if part or all of an MD is in the projection of any IO, the MD needs to be removed. Next, the MDs are compared with the buffer distance, and the shorter ones are defined as narrow gaps (Figure 7d). This process of calculation and comparison is repeated at each IO until all IOs are dealt with, at this point all narrow gaps are found. Figure 7e shows all MDs between the endpoints of each IO and the IOs in its buffer, and Figure 7f shows all narrow gaps in Room A. Another point worth noting is that the narrow gaps and the IOs produce several sealed areas (the areas surrounded by dash lines in Figure 7f). For the agent, the connectivity of these sealed areas is limited due to the narrow gaps. The procedures for the generation of sealed areas are provided in Appendix C. 

**Figure 9:**
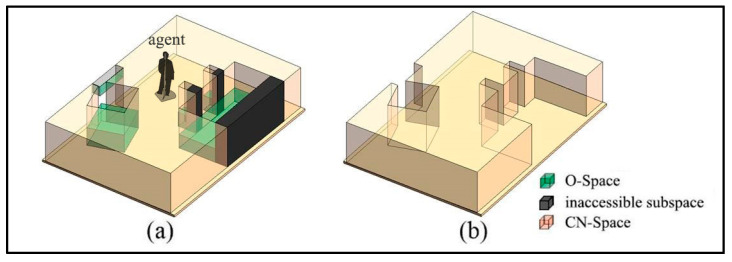
The CN-Space of Room A. (**a**) Inaccessible subspaces. (**b**) CN-Space.

Finally, CN-Space is generated by considering these obstacles and narrow gaps. In 3D space, the sides of IOs and narrow gaps are regarded as impassable walls, and these impassable walls re-divide the indoor space in the vertical direction. Sealed areas are extended conceptually to sealed subspaces. A sealed subspace is formed with a sealed area as the bottom face (0 and $H_{private}$ are set as its $H_{down}$ and $H_{up}$ respectively). The agent cannot move from one sealed subspace to another. Hence, considering the location of the agent, the subspace that the agent is in is selected as a reasonable navigable subspace. The other inaccessible sealed subspaces would be removed for the agent (Figure 9a). Besides, IOs and the subspaces above or under them are unsuitable for navigation, which is expressed as $S_{unsuitable}$. In this way, the special case shown in Figure 8 can be well resolved, since the sides of the IOs are also considered as impassable walls from the 2D plane to the 3D space. Therefore, the overlapping area is extended into a sealed subspace that is inaccessible to the agent. Lastly, considering the agent’s location, the CN-Space is represented by the remaining space after deleting all non-navigable subspaces from the whole indoor space (Figure 9b), which is expressed as:

(8) $$S_{CN}=S_{indoor}-IO-S_{h}-S_{os}-S_{unsuitable}$$

where $S_{indoor}$ is the whole indoor space and $S_{os}$ is other sealed subspaces except that the agent is located.

## 3. Use Case Examples

Two cases with real data collected from the Engineering Center Building at China University of Geosciences in Wuhan, China, were conducted to evaluate the effectiveness and advantages of the proposed approach. In this section, the FSS framework proposed by Diakité and Zlatanova [26] and the AccessVOR framework proposed by Yaagoubi et al. [38] are selected as the baseline methods for comparison. The FSS framework provides fundamental definitions (e.g., O-Space and F-Space) for our proposed method and is also an important fine-grained and context-aware subdivision framework of indoor spaces. The AccessVOR framework represents the latest research on indoor navigation considering users’ dimensions. The navigation network of the AccessVOR framework is built directly based on the Voronoi Diagram of indoor objects in the 2D plane and does not involve the generation of navigable space. Thus, our proposed method and the FSS framework are first compared and discussed from two aspects, the generation of F-Space (microscopic) and navigable space (macroscopic). Then, the comparison of the three methods in path generation is conducted to verify the adaptability of the proposed method to specific agents. 

**Figure 10:**
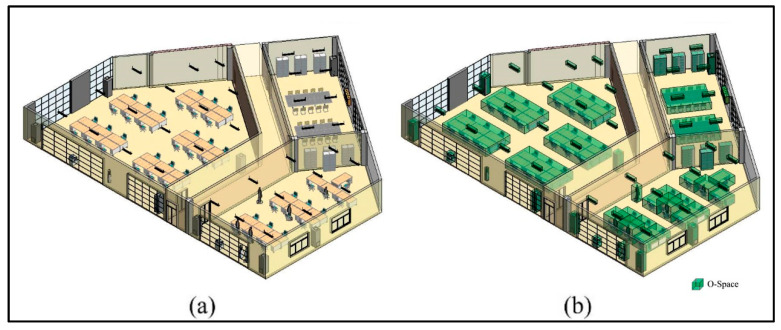
The layout of the experimental environment. (**a**) Actual indoor environment. (**b**) Simplified indoor environment.

**Figure 11:**
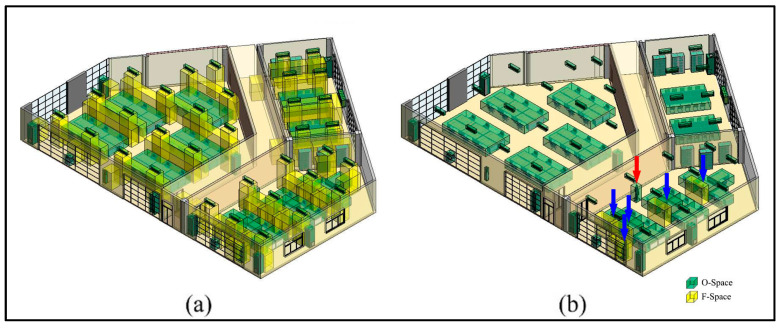
The layout of the experimental environment. (**a**) All F-Spaces of indoor objects. (**b**) Non-navigable F-Spaces of indoor objects.

The experimental environment is an office floor of the Engineering Center Building, which consists of three rooms and a corridor, and is expressed in the Building Information Model (BIM) (Figure 10a). These rooms are populated by regular indoor objects (e.g., tables, chairs, humans, lamps, and so on). First, to simplify the indoor environment, these indoor objects are aggregated (e.g., a table and a chair are combined as a workbench) according to the aggregation rules [26]. Then, O-Spaces of the indoor objects are generated to further simplify the indoor environment (Figure 10b). Next, F-Spaces of the indoor objects are calculated according to Equations (3)–(5) in Section 2.3 and an ordinary adult male with user dimensions 〈1.75 m, 0.53 m, 0.29 m〉. The dimensions come from the actual measurement of a male volunteer with normal physical characteristics. All F-Spaces of the indoor objects in the experimental environment are shown in Figure 11a. Figure 11b illustrates defined IOs including F-Spaces of the indoor objects interacting with their indoor users and O-Spaces of all indoor objects. Lastly, the CN-Space and navigable space are generated using our proposed method and the FSS framework, respectively.

A navigation system may serve multiple navigation agents, but it is worth noting that a single navigation subtask is only customizable to one specific navigation agent. When the navigation service is provided to a specific agent, the navigation system regards other agents who are also using the navigation services in the meantime as general indoor users. For example, in Figure 11b, the person marked by the red arrow is an agent of another navigation task who is not interacting with any indoor object and he/ she is treated as an indoor user in this case. In addition to him/her, the indoor users also include five people who are interacting with indoor objects and they are marked by the blue arrows. Therefore, based on the framework proposed in this paper, the six participants are all treated as general indoor users, and the rules of general indoor users are followed when dividing the indoor space.

To verify whether the algorithm could adapt well to the navigation agents with different dimensions, CN-Spaces are generated for a non-disabled adult and a disabled adult, respectively. 

### 3.1. CN-Space for a Non-Disabled Adult

In this subsection, a non-disabled adult male is considered an indoor navigation agent. Based on the dimensions 〈1.75 m, 0.53 m, 0.29 m〉 of the ordinary adult male, the O-Space of the agent is generated. Besides, according to the study of Nakauchi and Simmons [45], 0.4 m is considered a safe and comfortable distance and is set as the separation distance of private space. Thus, when calculating his private space, the agent dimensions are modified to 〈2.15 m, 1.33 m, 1.09 m〉.

**Figure 12:**
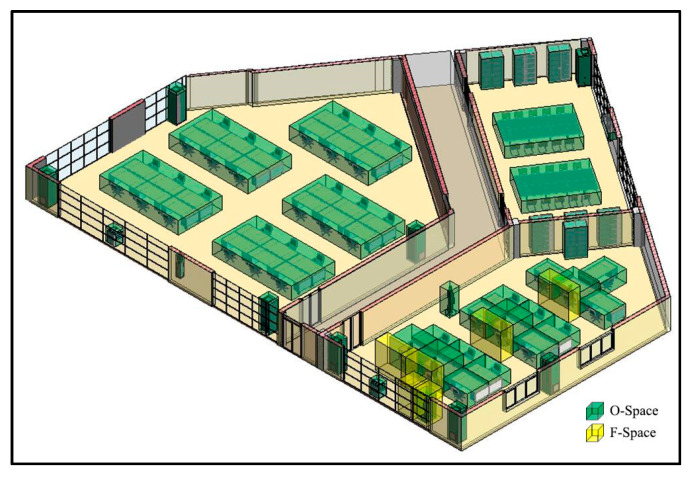
The indoor spaces that participate in the navigation for a non-disabled adult male.

**Figure 13:**
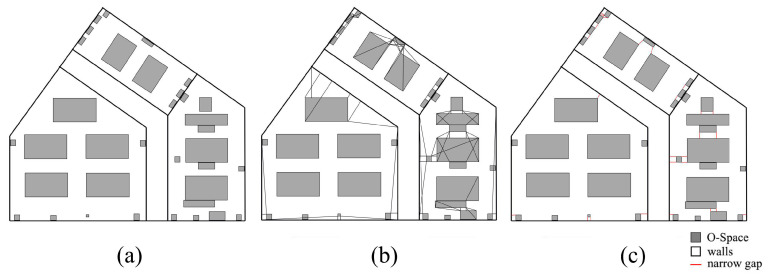
The generation of narrow gaps for a non-disabled adult male. (**a**) The projection of the experimental environment. (**b**) The MDs between each IO and every IO in its buffer. (**c**) The narrow gaps of the experimental environment.

**Figure 14:**
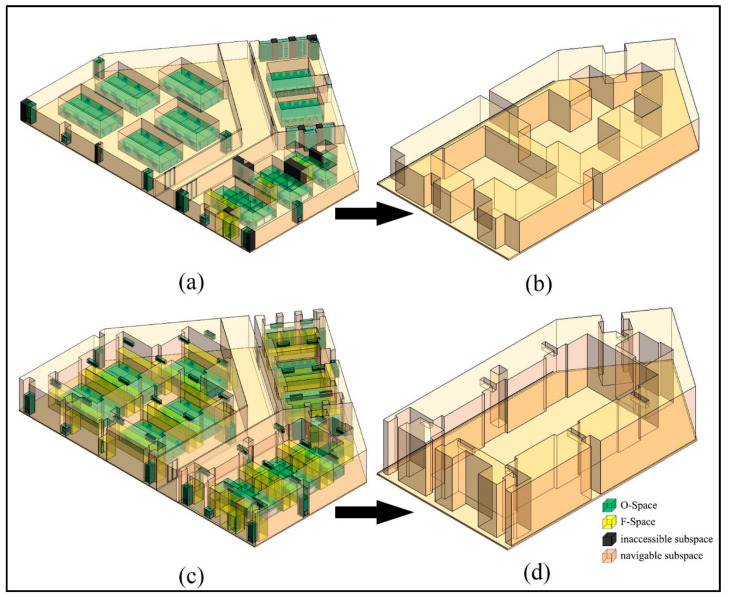
The differences between the generation of the navigable space between the proposed method in this paper and the flexible space subdivision (FSS) framework. (**a**) Inaccessible subspaces and CN-Space generated by the proposed method. (**b**) Partial detail presentation of CN-Space. (**c**) The navigable space generated by the FSS framework. (**d**) Partial detail presentation of the navigable space generated by the FSS framework.

Based on the above agent dimensions, the CN-Space of the agent is generated as follows. First, his $S_{h}$ is invalid in the vertical direction. A subdivision is conducted and spaces lower than 2.15 m are preserved (Figure 12). Next, in the horizontal direction, the remaining IOs are projected onto the floor (Figure 13a). The buffer distance is calculated to be 1.72 m based on Equation (7), namely $\sqrt{1.33^{2}+1.09^{2}}$. Taking a room or a corridor as a processing unit, the buffer operations are performed for each IO. In a processing unit, the MDs between each IO and other IOs in its buffer are calculated (Figure 13b) and all MDs less than 1.72 m are considered narrow gaps (Figure 13c). Then, the narrow gaps and the IOs produce several sealed spaces in the indoor environment. Suppose the agent is in the corridor and the sealed spaces that are unconnected to the corridor become impassable, where the CN-Space is the remaining space after removing all non-navigable subspaces from the entire indoor space (Figure 14a). The CN-Space in the lower right corner of the room is enlarged to clarify the details (Figure 14b). Meanwhile, the navigable space of the same building generated using the FSS framework is shown in Figure 14c. To clarify the details, the enlarged room in the lower right corner is also shown in Figure 14d. 

The difference between the navigable space generated by our proposed method and the FSS framework is analyzed from two aspects. From a micro perspective, the generation of F-Space differs in two ways. On the one hand, in the FSS framework, although a conceptual definition of the F-Space is given, actual operating rules for generating F-Space of indoor objects are missing. However, according to the three specific operating rules defined in our method, fine-grained space subdivisions could be easily established. On the other hand, in the FSS framework, F-Spaces are usually generated for indoor objects that cannot move by themselves but can be moved (e.g., furniture), not all indoor objects are considered to generate an F-Space (e.g., people) (Figure 14c). To meet fine-grained requirements, our proposed method fully considers the establishment of computable F-Spaces for all indoor objects. 

From a macro perspective, the FSS framework may ignore the actual needs of agents for navigable space. First, the FSS framework considers those subspaces that cannot affect the navigation behaviors of the agent as navigable subspaces (i.e., $S_{h}$). This could increase the spatial complexity of the whole indoor navigable space and the cognitive burden of the agent (compare Figure 14b,d). Second, F-Space generated by the FSS framework is not the preferred navigable subspace and if it is not a necessary element during navigation, it is usually non-navigable. Third, the FSS framework ignores the agent’s perception of the navigation environment and some inaccessible subspaces are treated as navigable subspaces due to the neglect of the agent’s dimensions (compare the dark gray subspaces in Figure 14a,c). This may result in a navigation path that has poor accuracy and agent satisfaction. However, our proposed method only considers the F-Spaces of those indoor objects that are interacting with their indoor users as IOs, and uses the agent’s dimensions to explore more accurate indoor navigable spaces. Furthermore, it provides the agent with personalized and customized navigable space, as well as appropriate navigation paths, thereby eliminating those inappropriate subspaces generated by the FSS framework that are unable to be navigated.

### 3.2. CN-Space for a Disabled Adult

**Figure 15:**
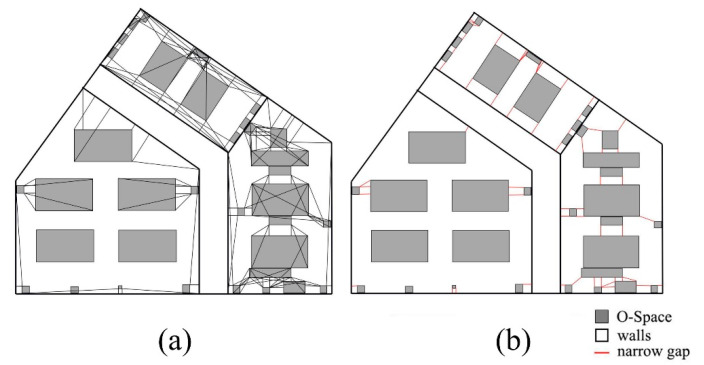
The generation of narrow gaps for a disabled adult male. (**a**) The MDs between each IO and every IO in its buffer. (**b**) The narrow gaps of the experimental environment.

**Figure 16:**
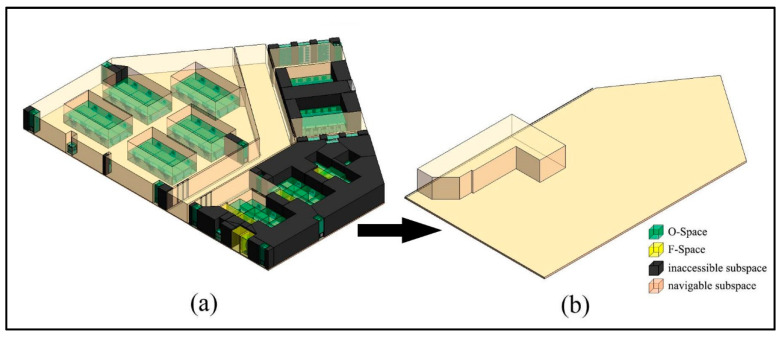
The CN-Space for a disabled adult male. (**a**) Inaccessible subspaces and CN-Space. (**b**) Partial detail presentation of the CN-Space.

In this subsection, a disabled adult male is considered an indoor navigation agent. This kind of agent usually moves with a wheelchair, so the agent’s O-Space is the space occupied by his body and the wheelchair. Here, the actual dimensions 〈1.55 m, 0.72 m, 1.2 m〉 of an ordinary disabled adult male are used and the separation distance of the private space is also 0.4 m. Thus, when calculating his private space, the agent dimension is modified to 〈1.95 m, 1.52 m, 2.00 m〉. Based on his dimension, the generation of the CN-Space for the disabled agent is similar to that of the non-disabled adult male. The buffer distance (2.51 m) of this case is greater than the previous case (1.72 m) in Section 3.1. Thus, the MD calculations become more complex because more MDs between the IOs need to be considered (Figure 15a). Then, the number of narrow gaps also increases, and more sealed spaces are formed than in the previous case (Figure 15b). Suppose the agent is in the corridor, the sealed spaces that are not connected to the corridor are impassable (Figure 16a). Note that CN-Space of this disable agent is just a sealed space near the door in the lower right room (Figure 16b). In fact, he could only move comfortably in a very limited space in the room. This indicates that the layout of the indoor objects in the room is unfriendly to the disabled agent. Thereby, a warning is suggested to be added at the entrance of the room.

Please note that the parameter of the separation distance could be adjusted in different scenarios. In this paper, the separation distance of the private space is set to 0.4 m to generate CN-Space. However, in emergencies, the separation distance can be reduced to 0.1 m, just to get enough navigable space for agents to leave dangerous areas quickly. Therefore, to leverage flexible parameter settings, the proposed approach can be used to establish navigable spaces for different purposes.

### 3.3. Generation of Navigation Paths

In this subsection, to verify the effectiveness of the proposed method, navigation paths are constructed for the same agent using our proposed method, the FSS framework, and the AccessVOR framework. The difference between the three methods will be examined to explore how the user dimensions affect the navigation path. 

The navigation network can be established by the navigable space. A common approach to derive a navigation network is to use the convex cell to subdivide the navigable space [18,20] and directly link the centroids of adjacent subspaces [13,26]. In this study, to further improve the accuracy and rationality of the navigation paths, the convex subdivision algorithm [46] is used to ensure the appropriate position of nodes (centroids) in the divided subspaces, and to increase the granularity level of the navigable space.

**Figure 17:**
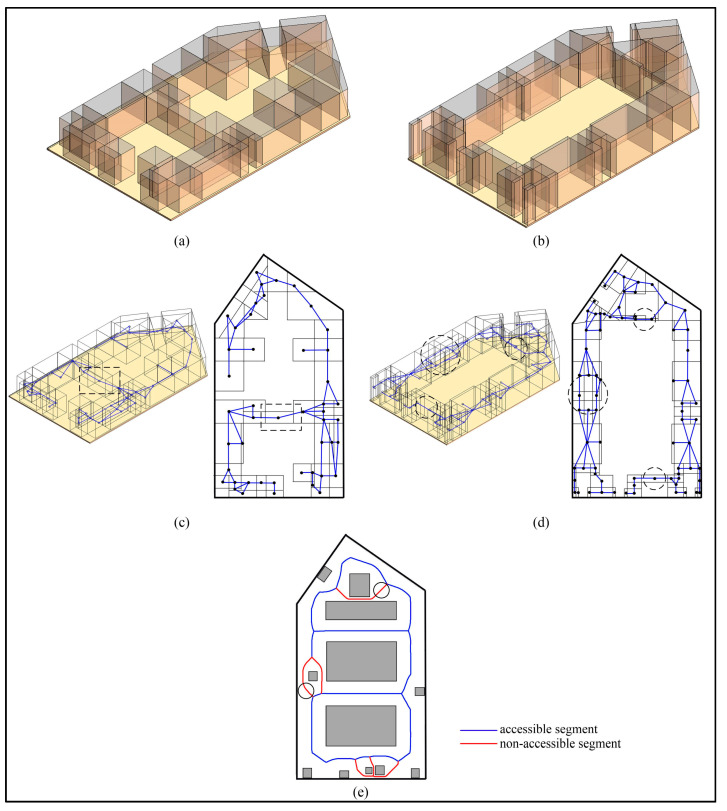
Generation of a navigation network. (**a**) Full convex subdivision of the CN-Space. (**b**) Full convex subdivision of the navigable space using the FSS framework. (**c**) Full connectivity graph of the CN-Space. (**d**) Full connectivity graph of the navigable space using the FSS framework. (**e**) The navigation network generated by the AccessVOR framework.

Take the above CN-Space of the non-disabled adult male in the lower right room as an example. Figure 17a illustrates the CN-Space using convex subdivision. A connectivity graph can be derived by linking the centroids of neighboring subspaces and is shown in Figure 17c, including a 3D view and a map view. For comparison, the navigable space of the same indoor environment using the FSS framework is performed using the same convex subdivision in Figure 17b. Figure 17d shows the connectivity graph generated by the same operation using the FSS framework. The navigation network generated by the AccessVOR framework is shown in Figure 17e. The accessibility of each segment is determined by comparing the agent’s dimension and the MDs between Voronoi edges and indoor objects.

The difference between the navigation networks generated by the three methods is analyzed from two aspects. From a micro perspective of the generation of F-Space, the generation of F-Space and its influence on the navigability of indoor spaces are not considered by the AccessVOR framework. Given the use of the FSS framework, the navigation network is only derived from the concept of F-Space, which limits the generation of F-Space and its further application in practice. However, the establishment of computable F-Spaces using our proposed framework for all indoor objects supports the feasibility of generating a fine-grained and context-aware navigation network. From a macro perspective of the actual needs of agents, first, only indoor objects on the floor are regarded as obstacles in the AccessVOR framework, ignoring other fine-grained spatial elements (e.g., furniture hung on walls or suspended in the air). This may result in a generated navigation network that is simple and unable to meet the agent’s needs for a comfortable and reliable navigation system. The navigable space generated by the FSS framework makes the navigation network over complicated, especially $S_{h}$ that cannot affect the navigation behavior of the agent may be subdivided into a large number of subspaces that have no practical use. To show the details more clearly, $S_{h}$ generated by the FSS framework have been removed from this example. However, our proposed method only considers the necessary fine-grained indoor spatial elements to reduce the cognitive burden of agents as much as possible. Second, all indoor spaces represented by the F-Spaces in the FSS framework and our proposed method are navigable in the AccessVOR framework because the functional features of indoor objects are ignored in the AccessVOR framework. Comparing Figure 17c,d, the FSS framework prevents the agent from passing those unused F-Spaces, which may result in the absence of some more convenient paths (e.g., the path in the black dotted box in Figure 17c) in the navigation network. While the judgment of the navigability of F-Space in our proposed method is more in line with the actual situation. Third, the agent’s dimensions are not considered in the FSS framework. Although the AccessVOR framework provides a solution to this problem, the granularity of its organizational units (Voronoi edges) is coarse, which may decrease the accuracy of navigability of indoor spaces. For example, the paths in the black circles in Figure 17e are not accessible when using the AccessVOR framework. However, the spaces represented by those paths are accessible in fact (see Figure 17c). In our proposed method, the subspaces that are not enough to get through breezily for the agent are removed from the fine-grained navigable space. This ensures that some inappropriate paths (e.g., the paths in the black dotted circles in Figure 17d) are removed from the navigation network, thereby not affecting the generation of reasonable paths. 

**Figure 18:**
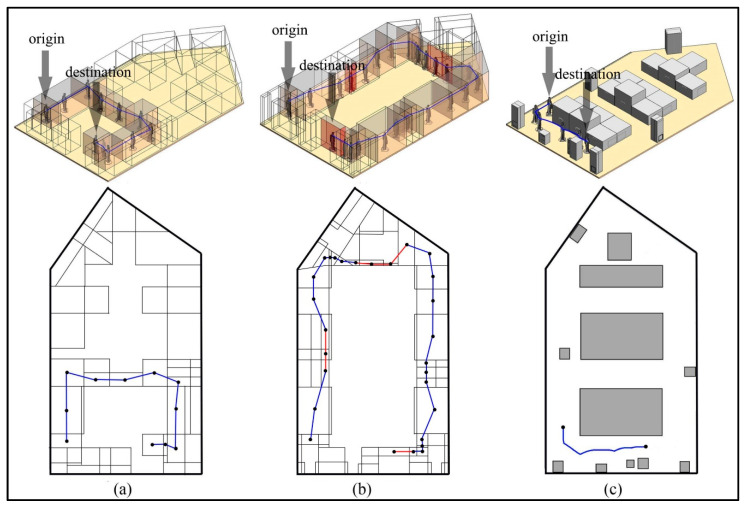
Generation of a navigation path. (**a**) Selected part of the connectivity graph for navigating the agent using the proposed method. (**b**) Selected part of the connectivity graph for navigating the agent using the FSS framework. (**c**) Selected part of the navigation network for navigating the agent using the AccessVOR framework.

The impact of these differences is quite evident in the generated navigation paths. Suppose the agent wants to get to his seat from near the door (see Figure 18). Based on the connectivity graph or the navigation network, a navigation path is generated between the origin and destination nodes according to the relevant shortest path algorithms (i.e., Dijkstra algorithm). Comparing Figure 18a–c, the navigation path generated by the AccessVOR framework is the shortest (13.38 m). However, the path guides the agent close to three active indoor users, which could be uncomfortable for them. The navigation path generated by our proposed method (35.58 m) is shorter than that generated by the FSS framework (69.41 m). Since our proposed method presents more suitable navigation subspaces with the rational use of unused F-Spaces, it will provide agents with more possible navigation paths that conform to the rules of human behavior. Although the navigation path generated by the FSS framework can reach the destination from the origin, it will pass through several blocked paths derived by narrow subspaces, which may not be suitable for the agent’s navigation (such as the red paths and subspaces in Figure 18b. These blocked paths account for 22.3% of the length of the entire navigation path, and the whole navigation path is divided into three parts, namely, 13.1%, 20.5% and 44.1%. This means that when the agent arrives at the blocked segment in the middle part of the navigation path, the path to the destination needs to be re-planned, and therefore affects the agent experience during navigation. These comparisons demonstrate that the proposed method in this paper considers users’ dimensions, optimizes the navigation network, and provides the agent with more reasonable navigable space and more comfortable and reliable navigation paths when compared with the FSS framework and AccessVOR framework.

## 4. Conclusion and Future Work

In this study, by considering users’ dimensions, a novel CN-Space generation approach is proposed to provide navigation agents with comfortable and reliable indoor navigation paths. First, according to the types of users (e.g., non-disabled, disabled), O-Spaces of users are analyzed and defined in detail. According to the functions, locations, sizes, and interactions of indoor objects, F-Spaces of indoor objects are determined. Those F-Spaces in use, all O-Spaces, and walls are regarded as indoor obstacles. Then, narrow gaps where agents cannot pass through easily are calculated based on defined indoor obstacles. Finally, a CN-Space generation method is proposed to achieve a comfortable navigable space by excluding inappropriate sealed spaces formed by indoor obstacles and narrow gaps from the entire indoor space. 

To verify the effectiveness of the proposed approach, two indoor navigation cases are conducted. Compared with the FSS framework and the AccessVOR framework, the proposed method has significant advantages and can provide agents of different dimensions with individual adaptive CN-Spaces and friendly paths in comfortable navigation environments. The proposed approach shows great potential for reducing the pressure of navigation agents, improving the comfort of navigation, and may play a key role, especially in unfamiliar indoor environments and emergencies. This research promotes the development of the FSS framework, from concept to realization, to enrich the connotation of the framework. Besides, this research extends the existing indoor navigation framework considering users’ dimensions from 2D space to 3D space, and elaborates the navigation network by combining the functional features of indoor objects and the agent’s perception of the indoor environment. It contributes to the enrichment of the theoretical and methodological body of knowledge for people-centric indoor navigation.

O-Space’s representation is the foundation of generating comfortable navigable space. Only preliminary methods to obtain users’ 3D O-Spaces are provided in this paper and the simplified spatial representation (cuboid) is a coarse geometric representation, which may make inaccessible free spaces useful. In future work, more studies on the optimal representation of O-Space need to be explored based on the relationship between human height and the O-Spaces for users of different age, race, and gender. 

## Appendix A. Generation of Updated IOs

**Algorithm** **A1:** Generation of Updated IOs.
**Input:** IO set *O*, Height of the agent’s private space *H_private_* **Output:** Updated IO set *UO* **Description:** * Bf(a) indicates that the operator is acquiring the bottom face of the object a.
1. *UO*← empty set(*ϕ*) 2. **if***O* is *ϕ* **then** 3. **return** *UO* 4. **else** 5. **for** $o_{i}\;\left(o_{i}\in O\right)$ **do** 6. **if** $H_{down}\left(o_{i}\right)>H_{private}$ **then** 7. *O*← *O*-{$o_{i}$} 8. **else if** $H_{up}\left(o_{i}\right)<H_{private}$ **then** 9. *UO*← *UO* U {$o_{i}$} 10. **else** 11. $o_{new}$← **new** *a cuboid* 12. $Bf\left(o_{new}\right)$← $Bf\left(o_{i}\right)$ 13. $H_{down}\left(o_{new}\right)$← $H_{down}\left(o_{i}\right)$ 14. $H_{up}\left(o_{new}\right)$← $H_{private}$ 15. *UO*← *UO* ∪ {$o_{new}$} 16. *O*← *O*-{$o_{i}$} 17. **end if** 18. **end for** 19. **end if** 20. **return** *UO*

## Appendix B. Generation of Narrow Gaps

**Algorithm** **A2:** Generation of Narrow Gaps.
**Input:** Projection set of walls *L* (line set), Projection set of other IOs *R* (rectangle set), Buffer distance *D_b_* **Output:** Narrow gap set *NG* **Description:** * C(a,b)=1 indicates that the object a is found in the buffer of the object b. * E(a) indicates that the operator is acquiring the endpoints of the object a. * MD(a,b) indicates that the operator is acquiring the MD between the object a and the object b. * L(a) indicates that the operator is acquiring the length of the object a. * IsIn(a,b)=0 indicates that the object a is not in the object b. * Ni(a,b) indicates that the operator is acquiring the number of the intersection of the object a and the object b.
1. *NG*← empty set(*ϕ*) 2. **if***R* is *ϕ* **and** *L* is *ϕ* **then** 3. **return** *NG* 4. **else** 5. **for** $r_{i}\;\left(r_{i}\in R\right)$ **do** 6. **if** $C\left(l_{k}\;\left(l_{k}\in L\right),r_{i}\right)=1$ **or** $C\left(r_{j}\;\left(r_{j}\in R,\;r_{j}\neq r_{i}\;\right),r_{i}\right)=1$ **then** 7. **for** $l_{k}$ **do** 8. **for** $E\left(r_{i}\right)$ **do** 9. *md*← $MD\left(E\left(r_{i}\right),l_{k}\right)$ 10. **if** L(md)<*D_b_* **and** $IsIn\left(md,r_{i}\right)=0$ **and** $Ni\left(md,l_{k}\;\left(l_{k}\in L\right)\right)<2$ **and** $Ni\left(md,r_{j}\;\left(r_{j}\in R\;\right)\right)<2$ **then** 11. *NG*← *NG* ∪ {md} 12. **end if** 13. **end for** 14. **end for** 15. **for** *r_j_* **do** 16. **for** $E\left(r_{i}\right)$ **do** 17. **if** $IsIn\left(E\left(r_{i}\right),r_{j}\right)=0$ **then** 18. *md*← $MD\left(E\left(r_{i}\right),r_{j}\right)$ 19. **if** L(md)<*D_b_* **and** $IsIn\left(md,r_{i}\right)=0$ **and** $Ni\left(md,l_{k}\;\left(l_{k}\in L\right)\right)<2$ **and** $Ni\left(md,r_{j}\;\left(r_{j}\in R\;\right)\right)<2$ **then** 20. *NG*← *NG* ∪ {md} 21. **end if** 22. **end if** 23. **end for** 24. **end for** 25. **end if** 26. **end for** 27. for *l_i_ (l_i_**∈**L)* **do** 28. **if** $C\left(r_{k}\;\left(r_{k}\in R\right),l_{i}\right)=1$ **or** $C\left(l_{j}\;\left(l_{j}\in L,\;l_{j}\neq l_{i}\;\right),l_{i}\right)=1$ **then** 29. **for** $r_{k}$ **or** $l_{j}$ **do** 30. **for** $E\left(l_{i}\right)$ **do** 31. *md*← $MD\left(E\left(l_{i}\right),r_{k}\right)$ **or** $MD\left(E\left(l_{i}\right),l_{j}\right)$ 32. **if** L(md)<*D_b_* **and** $IsIn\left(md,l_{i}\right)=0$ **and** $Ni\left(md,r_{k}\;\left(r_{k}\in R\right)\right)<2$ **and** $Ni\left(md,l_{j}\;\left(l_{j}\in L\;\right)\right)<2$ **then** 33. *NG*← *NG* ∪ {md} 34. **end if** 35. **end for** 36. **end for** 37. **end if** 38. **end for** 39. **end if** 40. **return** *NG*

## Appendix C. Generation of Sealed Areas

**Algorithm** **A3:** Generation of Sealed Areas.
**Input:** IO line set *IOL* (including the projections of walls *WL*, the sides of IOs’ projections *IL* and narrow gaps *NGL*), IO rectangle set *IOR* (the projections of IOs) **Output:** The sealed area set *SA* **Description:** * Uc(a) indicates that the operator is acquiring the usage counts of the object a. * Ci(a) indicates that the operator is cutting the intersecting lines in the object a to the generated new line set. * IsPY(a)=0 indicates that the object a is not parallel to the Y-axis. * Ar(a) indicates that the operator is acquiring the line with the smallest counterclockwise angle with the object a using the right endpoint of the object a as the vertex. * Au(a) indicates that the operator is acquiring the line with the smallest counterclockwise angle with the object a using the up endpoint of the object a as the vertex. * An(a,b) indicates that the operator is acquiring the line with the smallest counterclockwise angle with the object a using the endpoint of the object a that does not overlap with the object b as the vertex. * Ga(a) indicates that the operator is acquiring the area generated by all lines in the object a. * Fh(a) indicates that the operator is acquiring the rectangle in the object a. * Sf(a,b) indicates that the operator is setting the object a as a hole of the object b. * Mo(a) indicates that the operator is subtracting one of the usage counts of the lines that compose the object a.
1. *SA*← empty set(*ϕ*) 2. **if***IOL* is *ϕ* **then** 3. **return** *SA* 4. **else** 5. Uc(WL)← 1 6. Uc(IL)← 2 7. Uc(NGL)← 2 8. *NIOL*← Ci(IOL) 9. TS← **new** an empty set(ϕ) 10. **for** $nio_{i}$ *(*$nio_{i}$∈NIOL) **do** 11. **if** $Uc\left(nio_{i}\right)>0$ **then** 12. *TS*← *TS* ∪ {$nio_{i}$} 13. **if** $IsPY\left(nio_{i}\right)$=0 **then** 14. $nio_{j}$← $Ar\left(nio_{i}\right)$ 15. **else** 16. $nio_{j}$← $Au\left(nio_{i}\right)$ 17. **end if** 18. **if** $Uc\left(nio_{j}\right)>0$ **then** 19. $l_{old}$← **new** *a line* 20. $l_{old}$← $nio_{i}$ 21. $l_{new}$← **new** *a line* 22. $l_{new}$← $nio_{j}$ 23. **while** $l_{new}\neq \;nio_{i}$ **do** 24. *TS*← *TS* ∪ {$l_{new}$} 25. $nio_{k}$← $An\left(l_{new},l_{old}\right)$ 26. **if** $Uc\left(nio_{k}\right)>0$ **then** 27. $l_{old}$← $l_{new}$ 28. $l_{new}$← $nio_{k}$ 29. **end if** 30. **end while** 31. *a*← **new** *an area* 32. *a*← Ga(TS) 33. **if** ior (ior∈IOR)=Fh(a) **then** 34. Sf(ior ,a) 35. Mo(ior) 36. **end if** 37. **if** a∉SA **and** a∉IOR **then** 38. *SA*← *SA* ∪ {a} 39. Mo(a) 40. **end if** 41. **end if** 42. **end if** 43. **end for** 44. **end if** 45. **return** *SA*
